# Suicide attempts in French Polynesia during the era of COVID-19: a prospective analysis over three years

**DOI:** 10.1016/j.lanwpc.2023.100899

**Published:** 2023-09-04

**Authors:** Johan Sebti, Marguerite Serres, Valérie Calabro, Guochuan Emil Tsai

**Affiliations:** a Département de Psychiatrie, Centre Hospitalier de la Polynésie Française, BP 1640, Papeete 98713, Tahiti, French Polynesia; b Department of Psychiatry and Biobehavioral Sciences, UCLA School of Medicine, Los Angeles, California 90095, USA

**Keywords:** Suicide attempts, French Polynesia, COVID-19, Pandemic, Catastrophe, Insularity, Prevention, Mental health

## Abstract

**Background:**

Past studies in French Polynesia have identified suicide as a significant concern, with a measured annual incidence of 79.4 attempts per 100,000 population during 2008–2010. In response to the COVID-19 pandemic, a monitoring system was established to track and investigate suicide attempts (SA).

**Methods:**

A prospective study was conducted between April 2020 and March 2023, including all patients referred to the French Polynesia Hospital Center for SA. Demographic factors as well as clinical parameters were analyzed.

**Findings:**

During the study period, 895 SAs were registered and confirmed, with a crude annual rate of 106.7 events and the adjusted rate at 113.2 per 100,000 population. Substantial majority of SA happened in the island of Tahiti. Half of the subjects did not have psychiatric diagnosis. There was a significant increase in SA from year 1 to year 3, with young people (female more than male) particularly at risk, especially in Tahiti. The normalized incidence among females younger than 20-year-old was as high as 310.4 per 100,000 population.

**Interpretation:**

Our data revealed an overall 34.4% increase in SA in French Polynesia, with a striking 54.9% increase during the third year of pandemic. The last year’s record high incidence, is confirmed by increased activity on suicide hotlines, notably in Tahiti. A correlation between COVID exposure and suicidal behaviors, both at the individual and social level, is suspected with young female in Tahiti being the most vulnerable. These findings highlight the need for reinforced prevention and an efficient suicide monitoring system even after the public health emergency was declared over.

**Funding:**

The study is investigator-initiated without funding.

**Research in context**

**Evidence before this study**

The psychological consequences of natural catastrophes, such as the COVID-19 pandemic, have never been thoroughly described in relation to suicide. With scattered geography and limited resources, French Polynesia is a heterogeneous territory with diverse economic, cultural, and ethnic backgrounds, experiencing rapid social changes and gradually being exposed to environmental risks. Data collection systems and publications in mental health are limited, but they have already reported a significant vulnerability regarding suicidal behaviors, with a general risk estimated to be twice as high in comparison to mainland France.

**Added value of this study**

To our knowledge, this is the first article documenting the dynamics of suicide attempts in an isolated South Pacific territory during the three year period of COVID-19 pandemic. The findings not only provide valuable data both on the clinical characteristics of suicide attempters and on a demographic scale during COVID-19, and allow an update on previous data dating back to 2010. Moreover, special groups of vulnerability, such as urban, young people, and especially women, can be distinguished.

**Implications of all the available evidence**

Our results highlight a significant increase in suicide attempts during the COVID-19 pandemic, which is even more noticeable in comparison to previous data from 2010, and most pronounced at the third year. The high proportion of young attempters, as well as people with no psychiatric comorbidities, especially in Tahiti, illustrates a change of paradigm that confirms growing needs in suicide prevention. Geographical data shows important disparities among the archipelagos of French Polynesia and can serve as a basis for local prevention programs. Monitoring systems and prevention efforts have to be reinforced over the course of the next years if not decades.

## Introduction

French Polynesia, an Overseas Territory of France consisting of five archipelagos with a population of 279,750 in 2021, is often regarded as a heavenly paradise due to its natural beauty, which has been widely promoted by the media and travel agencies. Tahiti is the island where majority of population reside (189,277 in 2017). However, despite its idyllic reputation, this South Pacific territory faces significant social and environmental challenges in the past few centuries, COVID-19 included.

Polynesian society, like many other South Pacific nations, has undergone rapid and successive changes. These changes began with colonization by the English and French in the 18th century and continued with the establishment of the Pacific Experimentation Center, which is known for having conducted French nuclear tests from 1964 to 1996. This period brought promises of significant economic growth to the region but also led to a radical transformation in Polynesian society. Rapid Westernization, which is more pronounced with each new generation, has resulted in a split with the historic traditional way of life based on a subsistence economy. The socio-economic gaps within the population have been growing. In 2015, 26% of Polynesians lived below the low-income threshold (compared to 14% in metropolitan France), and the richest 10% earned on average 9 times more than the poorest 10% (compared to 3.6 times in metropolitan France).^1^

While the traumatic legacy of the nuclear tests continues to cast a collective shadow over the territory, climate change has exacerbated the environmental risks faced by its archipelagos, including the threat of rising sea levels and dying coral reef. With a healthcare system highly centralized on Tahiti, remote archipelagos are facing low medical demography and limited local access to mental health services while the emergency mental health service has always been provided by French Polynesia Hospital Centre (Centre Hospitalier de la Polynésie Française, CHPF) alone. And the remote clinic and public health nurse will transport the people with mental health and suicide crises to CHPF, mostly by flight.

Past studies on the general population in French Polynesia suggest a disproportionate prevalence of mental disorders.^2^ Suicidal behaviors, in particular, are a rising concern, with a risk in the general population that has been estimated doubles that of mainland France (26% vs 13%), contrasted by a lower mortality rate (10.6 per 100,000 inhabitants).^3^ However, this phenomenon, described as the "suicide paradox",^4^ remains to be confirmed, with the latest published epidemiological data on completed suicide dating back as far as 2018.

**Fig. 1:**
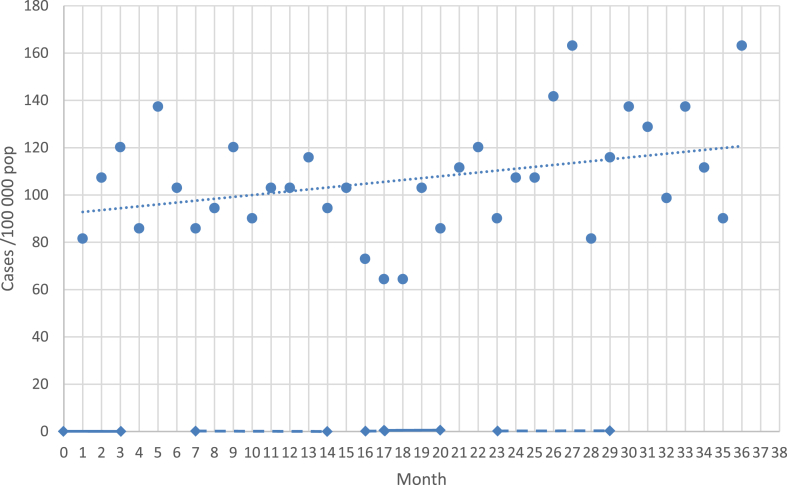
**Recorded incidence of suicide attempts during pandemic in French Polynesia.** For illustration: Solid lines indicate full lockdown periods, dotted lines indicate partial lockdown or social distancing measures only (source: Haut Commissariat de la République Française en Polynésie). Month 1 corresponds to April 2020, and month 36 corresponds to March 2023. Corresponding slope equation is Y = 0.1852X + 21.43492.

Climate change has been burdening the Pacific oceanic countries who are lacking the resources and technology while facing the blunt of its grave impact. From worse to worst, amidst this fragile environment, the World Health Organization (WHO) declared a public health emergency of international concern in January 2020 due to the widespread outbreak of COVID-19, which was later reclassified as a pandemic on March 11, 2020. The pandemic has brought a “double whammy” threat to the Oceanian countries, French Polynesia is no exception. With limited medical supply, vaccine and medicine initially, the health care system was overwhelmed and the casualty was high. Not to mention the disastrous socioeconomical impact, the catastrophe brought 2.3 death per thousand population of French Polynesia, comparable to the rate of 2.51 per thousand population in heavily inflicted France (https://ourworldindata.org/covid-deaths?country; May 2023). To date, French Polynesia has witnessed four distinct outbreaks of COVID-19, with the deadliest being the "delta" wave from July to October 2021, responsible for the majority of deaths and severe cases, and which challenged the healthcare system to its limits. This event of unprecedented magnitude in the 21st century had significant social and economic implications, particularly due to the implementation of social distancing and lockdown measures in many regions of the world (Fig. 1). Globally, the psychological aftermath of the pandemic remains closely monitored, with particular concern for vulnerable populations.

In French Polynesia, a territory known for its strong insularity, rampant socio-economic disparities, and challenges in accessing mental health services, the rising risk of mental health, in particular suicide was a major concern amidst the COVID-19 pandemic. In response to the first reported cases of COVID-19 in the territory, our team conducted a study to track all suicide attempts (SA) during COVID-19 by applying a very similar, if not identical, methodology as the study done in the previous decade.^5^ This also serve as an excellent reference to understand the evolution of suicide behaviors.

In this article, we report the findings obtained from the first 36 months of monitoring, between April 2020 and March 2023 which coincide the time period of public health emergency of COVID, to understand the impact of the pandemic disaster on SA behaviors in French Polynesia. We characterized all registered SA by analyzing the demographic and clinical characteristics, and compared our findings with the previous study for the period of 2008–2010^5^ which was conducted in the exact same setting at French Polynesia and measured an incidence of 79.4 SA per 100,000 inhabitants, which was later estimated to be as close as 98 per 100,000 inhabitants if considering potential missed-out subjects.

We hypothesized that the COVID-19 pandemic may contribute to an exacerbation of suicidal behaviors, thereby increasing the overall human toll, while acknowledging a notable level of uncertainty regarding the temporal progression of these events. In Australia, France, and the US, the suicide rates, in fact, remained unchanged or even dropped during the early phase of COVID-19.^6^, ^7^, ^8^ We attempted to investigate whether we would observe a similar trend in French Polynesia or if there would be a double whammy effect when a catastrophic disaster like COVID perpetuated in the remote society of French Polynesia.

## Methods

We conducted a prospective longitudinal naturalistic study, including all patients admitted to the emergency service at CHPF following a SA between April 1st, 2020 and March 31st, 2023. The CHPF, located in the suburban area of Papeete in Tahiti, is the sole healthcare facility with a psychiatry team available 24/7. As a result, almost all patients treated for SA behavior in French Polynesia has been transferred to CHPF for psychiatric evaluation, including those from remote archipelagos. This centralized mental health system facilitates the inclusion process and ensuring a recruitment of highly representative, if not all, population with SA. Follow up contact were made with community health centers in archipelagos to check for any missed-out case of SA that was not transferred to CHPF, and thus were labeled as “missing data” case when inclusion from the CHPF was not feasible.

The SA was defined according to the criteria of WHO/EURO group^9^: a self-initiated behavior aimed at the intention to die, which may or may not result in physical injuries, including parasuicide. The SA behaviors were also defined by the same criteria in the study of comparison.^5^ We follow the definition of NFSB with the exception to completed suicide, patients with isolated suicidal ideation, impulsive or accidental self-harming behaviors without suicidal ideation attested by the patient or families, or any patient with missing data.

Information was collected from the initial semi-structured clinical interview with predetermined theme and the patient's medical record for complementary purposes after informed consent was obtained from the study participants. The investigation team was composed of two research psychiatrists (JS and MS) and two research nurses. After medical and psychiatric evaluation, data were collected and anonymized by a psychiatry healthcare professional trained to the study, and processed monthly. The study protocol has been approved by the Ethical Board of the CHPF.

The main study variables were month and year of SA during the study period, gender and age, geographic place of stay at the time of the SA, self-harm method, and current or past psychiatric diagnosis. In case of multimodal SA, we retained the first method in chronological order. Diagnoses were established based on clinician’s interview and ICD criteria at the time of evaluation, or on past medical records if none was found during interview. If multiples diagnoses were present, we retained the most recent one. In our study, we also considered that a secondary hospitalization in psychiatry (excluding initial monitoring in the emergency department) reflected a severity criterion of the suicidal act or an increased risk of recurrence, and thus would be a variable of interest. Suicide methods were categorized into fifteen items, based on the definitions of the ICD-10. Associated diagnoses were grouped into eight categories, also based on ICD-10 criteria.

### Data analysis

Crude annual rates were calculated using the 2021 general population count of 279,544 from the Statistical Institute of French Polynesia (Institut de la Statistique de la Polynésie Française, ISPF). Standardization for specific age and geographical subgroups was based on the latest demographical data available from the 2017 census of ISPF. We tested the *a priori* hypothesis that the rate of SA increased during the COVID-19 pandemic, using the number of SA recorded per month. Year to year comparison were made by repeated ANOVA with post-hoc *t*-test and Bonferroni correction. Regarding the whole study period, deviation from horizontal trend was tested with a linear regression, time was considered the independent variable, and number of SA/month the dependent variable.

Secondary analyses were conducted to explore the characteristics of the SA during the study period at the individual level, with subgroup comparisons carried out with the two-sided Fischer exact test with 95% IC. Due to the nature of the study design and the specific research questions, correction for multiple comparisons of the secondary analysis was not carried out. However, when considering all tests as equal and applying Bonferroni correction, the alpha level would be set at 0.00714 (0.05/7).

All statistical analysis were conducted with R software v4.22.^10^

### Role of the funding source

No funding was received.

## Results

During the study period, a total of 895 SAs were registered and confirmed based upon the inclusion criteria, involving a total of 764 individuals. Fifty-eight events were excluded from the sample as they met exclusion criteria (4 completed suicides, 24 suicidal ideations without SA, 19 accidental self-harming behaviors or without suicidal ideation, and 11 SAs with missing data from the local medical team for SA not evaluated at CHPF). The corresponding rate of SA over the 36 months period was 320 events per 100,000 inhabitants (102.6, 94.6, 123.0/100,000 over the three study years from 2020 to 2023 respectively), which translates to a crude annual rate of 106.7 SAs per 100,000. Fig. 1 illustrated a significantly raising rate of SA during the study period.

### Demographics and geographic characteristics

10–19 and 20–29 yo age groups represented majority of the subjects (49.3% of SAs). The mean age was 33.0 years old (min 10 years, max 80 years, SD = 14.25), with a median of 30.2 years. The most represented age group was between 20 and 29 yo (30.0% of SA), with a gradual decrease in incidence throughout life. We registered 484 SA by females and 411 by males, with a gender ratio slightly favoring women (54.1%). The gender difference for the youngest age group is prominent (<20 yo, p < 0.01), but with no significant differences throughout the rest of life span (Fig. 3).

**Fig. 2:**
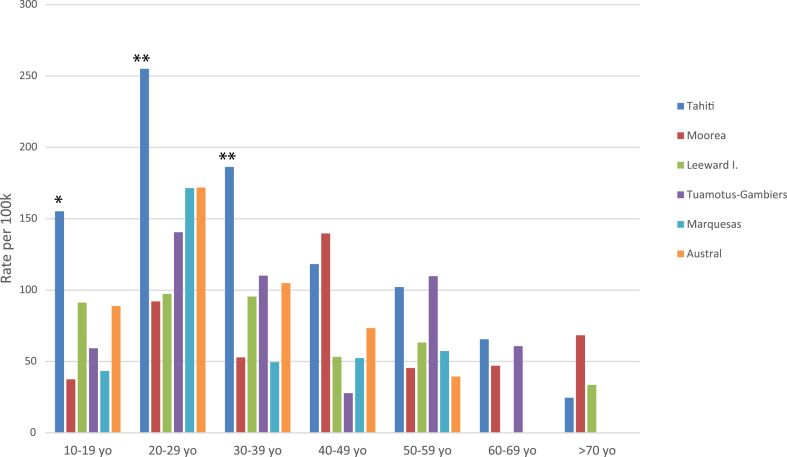
**Standardized rate of suicide attempt by age and locality.** Statistical significance was tested by comparing the subgroup rate vs overall rate for the whole cohort. ∗∗p < 0.01, ∗p < 0.05.

The vast majority of SA (81.6%) took place in the most populated island of Tahiti, followed by the Leeward Islands (Raiatea, Bora-Bora, Huahine, Maupiti; 7.9%), Moorea (7.2%), the Tuamotus-Gambiers (4.0%), the Austral Islands (1.6%), and the Marquesas (1.7%). 0.4% of patients had no determined geographical origin or were in transit in the territory (Table 1 and Fig. 2).

**Table 1:** Geographic distribution of suicide attempts.

Place of residence	N	%
Tahiti	730	81.6
Leeward Island	64	7.2
Tuamotus-Gambiers	36	4.0
Moorea	32	3.6
Marquesas Islands	15	1.7
Austral Islands	14	1.6
Other/non specified	4	0.4
**Total**	895	100.0

### Time course of SA

Throughout the three-year duration of the study, we observed variations in the average number of SA monthly. The Repeated Measures ANOVA test (df = 2, df Total = 11, df Error = 9) indicated that there was a significant difference in the monthly number of SA between the different years, F(2, 22) = 6.9, p = 0.0047, with a mean SA number per month of 23.92 for year 1, 22 for year 2, 28.67 for year 3. The post-hoc paired t-test test, applying a Bonferroni corrected α = 0.017 indicated that the means of the following pairs were significantly different: y_1_–y_3__(F = 8.13 p = 0.015_, y_2_–y_3__F = 12.25 p = 0.004__)_. The observed effect size η^2^ is large, 0.26. The partial effect size η_p_^2^ is 0.39.

Furthermore, when exploring the relation between the number of SA per month to the time of the study (36-months period), linear regression found a statistically significant positive deviation from a horizontal trend (R^2^ = 0.12, F(1, 34) = 4.7, p = 0.037. β = 0.19, α = 21.43, p < 0.001), suggesting a significant upward trajectory in SA rates (Fig. 1). Corresponding slope equation was Y = 0.1852X + 21.43492.

### SA method

Voluntary drug intoxication remained the most represented method of suicide (56.8% of the SA), followed by hanging (19.9%), phlebotomies (6.8%), and SA with sharp or penetrating objects (3.0%) (Table 2). The difference between men and women was uniquely significant for hanging (27.3% of total SA in men vs 9.5% in women, OR = 4.49, CI [3.08; 6.65], p < 0.001) and intentional drug intoxication (68.8% of SA in women vs 36.2% in men, OR = 0.33, CI [0.25; 0.44], p < 0.001).

**Table 2:** Distribution of suicide method.

Suicide method	Total		Male		Female		Hospitalization	
	N	%	N	%	N	%	N	%
X60–65—Medications and drugs intoxication^a^	508	56.8	175	36.2	333	68.8	181	35.6
X66, 68–69—Chemicals intoxications	26	2.9	11	2.3.	15	3.1.	9	34.6
X67—Exposure to gases or vapours	3	0.3	1	0.2.	2	0.4.	1	33.3
X70—Hanging^a^	178	19.9	132	27.3	46	9.5.	71	39.9
X71—Drowning	7	0.8	5	1.0.	2	0.4.	4	57.1
X71—Strangulation	17	1.9	8	1.7.	9	1.9	6	35.3
X76–77—Immolation or burning	7	0.8	4	0.8.	3	0.6.	1	14.3
X78—Cutting (wrist)	61	6.8	27	5.6	34	7.0.	21	34.4
X78—Cutting or piercing (other)	27	3.0	13	2.7.	14	2.9.	12	44.4
X78—Cutting (neck)	3	0.3	2	0.4.	1	0.2.	0	50.0
X79, X83–84—Other method	8	0.8	5	1.0.	3	0.6.	4	42.9
X80—Falling	27	3.0	16	3.3.	11	2.3.	11	40.7
X81–82—Vehicles or moving objects	23	2.6	12	2.5.	11	2.3.	4	17.4

a p < 0.001.

Also, SA with physical force, such as hanging and cutting appears as a statistical trend of choice among the 20–29 years old vs other age subgroups (OR = 1.33, CI [0.99; 1.79], p = 0.05).

### Associated psychiatric diagnoses and hospitalization

Table 3 sums up the main findings regarding distribution of psychiatric diagnosis among the SA subjects. In 49.6% of cases, no current or past psychiatric diagnosis was ascertained. The proportion of patients with no diagnosis was at his maximum for younger age classes (63.9% for 10–19 yo), before slightly dropping by age groups. When a diagnosis was present, it mainly consisted of a history of isolated suicidal behavior (X60–84 in ICD10, 15.3%), depressive episodes (9.9%), and psychotic disorders (7.9%). There was a higher ratio of F20–29 psychotic disorder in male (OR = 6.91, CI [3.52; 14.82], p < 0.001) and more F40–48 anxiety and neurotic disorders in female subjects (OR = 0.19, CI [0.04; 0.58], p < 0.001). No significant difference was found between men and women in the rest diagnoses.

**Table 3:** Diagnosis associated with the suicide attempts.

Psychiatric disorder	Total		Male		Female	
	N	%	N	%	N	%
F20–29. Psychotic disorders	71	7.9	57	13.9	14	2.9
F31. Bipolar disorder	24	2.7	13	3.2	11	2.3
F32. Depressive disorder	89	9.9	36	8.8	53	11.0
F40–48. Anxiety and neurotic disorders	27	3.0	4	1.0	23	4.8
F60. Personality disorders	61	6.8	29	7.1	32	6.6
X60–84. Self-harm	137	15.3	52	12.7	85	17.6
Other diagnoses (including substance-related or addictive disorders)	42	4.7	24	5.8	18	3.7
No diagnosis	444	49.6	196	47.7	248	51.2

Overall, 325 SAs (36.3%) were followed by a hospitalization in psychiatric inpatient ward after initial monitoring in the emergency service. This rate increased to 52.7% when the subjects carried psychiatric diagnosis. The hospitalization was notably more frequent for youngest patients (44.7%) or those with psychotic (69.0%) or bipolar (58.3%) comorbidities (table not shown).

### Repeated suicide attempts

SAs were separated into two sub-groups, one for SA with only one subject-Id and the other for SAs with recurrent subject-Id, in order to allow a comparison based on group of individuals rather than group of events (SA). The “unique attempter” group was composed of 676 subjects. 132 SA events were identified as recurrent SA during the study period, involving 87 individuals (14.7% of the SA, 11.3% of the individuals). We found one individual with 17 cumulative SA, one with 10 SA, one with 6 SA, one with 5 SA, three with 4 SA, nine with 3 SA, and seventy-one with 2 SA.

The mean time to recurrence of a SA was 193 days (SD = 235 days), with a median of 91 days. This particular subgroup of “SA repeaters” was characterized by a similar gender ratio (47 females and 38 males) and age distribution (mean age = 34, SD = 14). 81% of them were from Tahiti, also comparable to the whole cohort. Psychiatric diagnosis was found for a slightly higher proportion (61.1%), in comparison to the main sample at 49.6%, with no statistical significance. Referring to unique individuals, The most common diagnosis at the time of the first recorded SA were past history of SA (26.4%, OR = 2.52 CI [1.41; 4.39] p < 0.001; Two-sided Fisher’s Exact Test)) and depressive disorders (12.6%, not significant). Most frequent suicide methods were autointoxication with drugs (64.4%) hanging (18.3%), and ingestion (4.6%), all being not significantly different from the general cohort. 47 of them (54.0%) applied the same method for following SA.

### Standardized rates by age and geographical area

Considering the highly heterogeneous environment and relative geographic isolation of the islands, spread in an oceanic area the size of Europe, we hypothesized that incidence rates for SA would be more representative if adjusted to the corresponding age group and geographic locality. To investigate this possibility, the number of registered SA was standardized with the available population data for each geographic group, and the crude SA rate of 106.7/100,000 was used as a comparison basis.

**Fig. 3:**
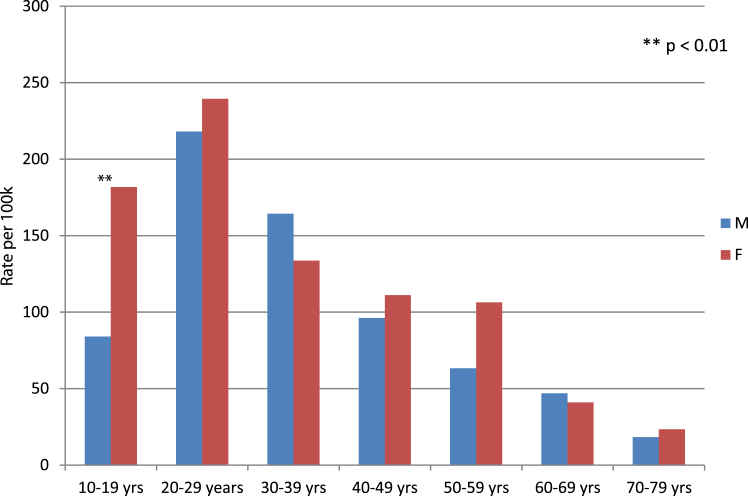
**Standardized rate of suicide attempt by age andgender.**

After adjusting for geographical place of residence, Tahiti had the highest annual incidence of SA at 126.23/100,000 pop compared to an average incidence rate of 62.1/100,000 pop for the other islands. Regardless of the place of living, age normalization shows that SA incidence was notably higher for groups under 40 years old, with a peak value of 229.9/100,000 population for the 20–29 yo. Among individuals aged 10–19, we noted a significant gender gap in the incidence of SA, with young women having almost twice the incidence rate of young men (181.7 vs 84.0/100,000 population) (Fig. 3). These findings are even highlighted when applying a second layer of normalization with both age and locality, with a concerning rate of 255.0/100,000 for the 20–29 yo in Tahiti. Assuming an equal gender repartition, teenagers and young women between 10 and 20 yo in Tahiti reached the maximum standardized incidence with an estimate of 310.4/100,000 SA per year.

In addition, the Tuamotus-Gambiers, Marquesas and Australs archipelagos displayed higher incidence rates, for individuals aged 20–29 (140.5; 171.4 and 171.8/100,000). Incidences were also higher for 40–49 yo in Moorea (139.7/100,000) and for 50–59 yo in Tuamotus-Gambiers (109.7/100,000), although these differences were not statistically significant due to the limited sample size.

## Discussion

We investigate the behaviors of SA in French Polynesia and characterized the incidence in time, main demographics and clinical characteristics of the study subjects admitted to the emergency service at CHPF following their SA during the three years of public health emergency of COVID-19. To our knowledge, this is the first study to provide a prospective detailed analysis of SA in a single country encompassing the whole COVID-19 pandemic era.

Our study environment of French Polynesia is unique in its highly concentrated mental health service in a single institution where most of the mental health providers in the country practice. And most of both the reported and self-referral subjects at French Polynesia who developed SA were evaluated and treated in CHPF. The health care system therefore provides a reliable setting to investigate the SA systemically and comprehensively with continuity for French Polynesia as a whole. Further, we were able to investigate the chronological variation of SA not only during the COVID era of three years (so far), but we also can fairly compare to the study by Amadeo et al.^5^ with the period of 2008–2010, since both studies were conducted in the same setting, and with almost the identical methodology.

SA represents a grave mental health consequence when a vulnerable society facing catastrophe like pandemic of COVID-19. We found a higher crude incidence of SA (106.7/100,000) compared to the previous study period,^5^ which reported a rate of non-lethal suicide behaviors estimated at 79.4/100,000 inhabitants from a similarly acquired sample of subjects in French Polynesia during pre-pandemic years of 2008–2010. During the same timeframe of our study, the Non-Governmental SOS Suicide hotline recorded a 66.2% increase in the global number of crisis phone calls from 947 calls of 2019, 804 calls of 2020, 1113 calls of 2021, to 1574 calls of 2022 (SOS Suicide annual activity reports, personal communication), which corroborated the increase of recorded SA during the COVID-19 years we observed. Also, the incidence will be higher at 113.2/100,000 if we include subjects not transferred to CHFP by the local clinics/hospitals (n = 11), with the isolated suicidal ideation (n = 24) and including the patients who openly and consistently denied any suicidal intent (n = 19). The general conclusions would still be unaffected if we included these subjects, while the total volume of patients admitted to emergency rooms (for all causes combined) in CHPF remains relatively stable over the 2008–2023 period (46,721–43,149 of 2009–2011; 45,902–44,978 of 2019–2021, CHPF activity report, personal communication).

The incidence increased over the last decade highlighted by our study is substantial. Although there is no study report of SA in the interim between 2010 and 2019 and it is not possible to rule out a gradual increase over the last decade, our findings align with other studies that have reported an overall higher incidence rate of suicidal behavior during the pandemic, particularly among young individuals.^8^^,^^11^ At the same time, although not being reported after 2017, the number of death by suicide in French Polynesia has been continuously decreasing from 2005 to 2017 (17.2/100,000 pop during the 2005–2010 period vs 10.6/100,000 pop in 2017.^12^ Given SA is a high predictive risk factor for completed suicide, a progressive increase in SA in the interim period is highly unlikely.^13^ Thus, we may first hypothesize that this increase is linked to the exacerbation of biopsychosocial difficulties caused by the catastrophic pandemic infection as well as the prolonged confinement measures and reaction to stress within an isolated territory which economy is largely dependent on tourism.

Within the 36 months period of the study, we also recorded a significant increase of SA in time (Fig. 1). Different from the hope that the incidence of SA would have been abating gradually by adaptation to the pandemic stress and/or by more service and help provided by the government, the third year revealed a much higher rate of SA compared to the first and second year of pandemic (Fig. 1). It is unclear how long this trend will sustain after the declared public health emergence of pandemic was declared over officially. It is nevertheless a warning sign that suicidal behavior can perpetuate beyond pandemic. It calls for a much needed mental health prevention and service during the “recovery” from catastrophe like COVID-19.

An alternative perspective for analysis involves considering the first year of the study as a period close to the pre-COVID state in French Polynesia, given the delayed outbreak consequences while increase of suicide events during COVID has not been reported consistently.^14^ Although the first cases of COVID-19 were recorded in March 2020, the number of cases and deaths of COVID-19 remained relatively low during strict social distancing measures and an active lockdown. As a result, some immediate psychological consequences of the outbreak may have been mitigated, and SA rates could be comparable to pre-pandemic levels like a volcano turn active right before eruption. The small and insignificant decline observed in the second year of the study (April 2021–March 2022) aligns with findings reported in other countries, which indicate that SA rates and suicide mortality tend to decrease during the acute phase of an outbreak, followed by a subsequent rebound.^8^ In fact, analyses of suicide rates during the COVID-19 based on large scale health statistics from across the world report either a modest reduction in total suicide rates or no significant net increase in suicide rates during the early year of the COVID-19.^6^^,^^7^ The overall trend of suicide hotline records discussed above also were consistent with this scenario.

Alternatively, the first two years of COVID-19 were devastating enough and the third year’s dramatic increase in SA, 54.9% increase compared to 2008–2010 and a 30.0% increase from the year before, represents a gravely concerning trend of the psychological aftermath of the pandemic that will sustain, at least for the near future years. Since August 1, 2022, the travel restrictions in effect between French Polynesia and mainland France have been lifted. While French Polynesia experienced relative political stability and progressive economic growth, mainly based on tourism income over the past decade, Gross Domestic Product drastically decreased from 2019 to 2020 after the initial lockdown, resulting in a negative growth index of −7%. The decline in income resulted in numerous households falling below the poverty line, possibly worsening their socio-economic vulnerability to the catastrophe of COVID-19. This finding calls for vigilance in the recovery planning to address the global psychological trauma that cannot be ignored or denied in the coming years.

French Polynesia has a much higher rate than the rest of Oceanian countries for COVID-19 mortality; French Polynesia 2.3, New Caledonia 1.08, Fiji 0.95, Australia 0.76, Samoa 0.76, New Zealand 0.43, Solomon Islands 0.21, Papua New Guinea 0.07, Vanuatu 0.04, per thousand population (https://coronavirus.jhu.edu/data/mortality; May 2023). Most of the mortality of COVID-19 in French Polynesia were from Tahiti. With the most-dense population and activities in French Polynesia, Tahiti was the most impacted island during the multiple COVID-19 outbreaks, which was also reflected in SA occurrences in the geographic distribution of our study samples (Fig. 2). In our study, 81.5% of the SAs happened in Tahiti island. It is important to note that in the previous study, which included 556 SAs,^15^ only 67% of them were from Tahiti (OR = 0.46, CI [0.35; 0.59] p < 0.001). In another words, COVID-19 exposure appears to be concentrated with SA occurrences within the geographic sphere we studied, without excluding the role of possible confounding factors, like modernization in the past decade in Tahiti. Neither the high concentration of SA in Tahiti can be explained by modifications of detection and referral systems nor the public health infrastructures in Tahiti, which were largely not changed in the past decade. Whether the geographic distribution in SA parallel to the mortality of COVID-19 in the rest of the world remains to be confirmed.

When the geographic of Tahiti island compounded by the vulnerability of young age, the rate of SA is strikingly high at 229.0/100,000 population for the 20–29 yo. In fact, vulnerable groups, such as youth, have been identified as particularly at risk due to societal changes, lack of prospects, isolation, and the deleterious role of social network.^16^ The mean age was 33.0 years old in the study cohort and we confirmed specific age groups of vulnerability, such as youth, display greater risk level, with adjusted incidence that can reach as high as 255.0/100,000 population, twice as high as those in their forties and older. Also, younger age was also more closely associated with the use of physical force in SA and secondary hospitalization in psychiatry. This appears to be particularly measurable in the most modernized Tahiti, which displays double the standardized incidence of SA compared to other islands (126.2/100,000 vs 62.1/100,000 respectively (Z-stat = 5.04; p = 0.001). Moreover, the striking difference between male and female teenagers (10–19 years old) raises questions about a particular vulnerability in young women (Fig. 3).

Voluntary drug intoxication remained the most represented method of suicide and hanging is the second most common method for all. Hanging happened about three times more frequently in male than female (Table 2). In remote island, hanging accounts for 28.6% of suicide attempts (vs 17.9% in Tahiti; OR = 1.82, CI [1.20; 2.71] p = 0.003). Autointoxication, in contrary, seems less common in remote island in comparison to Tahiti (47% vs 59% respectively, OR = 0.62, CI [0.43; 0.89] p = 0.007). Also, SA with the use of physical force, such as hanging and cutting appears as a statistical trend of choice among the 20–29 years old vs other age subgroups. In France, the dominant ways of SA are auto-intoxication with drugs or other toxics and injuries with sharp objects^17^ while hanging only occurs in 2.4% of recorded cases. Due to its widespread availability, hanging is likely perceived as a convenient and effective method,^18^ particularly in islands other than Tahiti where the access to alternative means (medications, vehicles) are more challenging. Moreover, cultural and gender-bound factors are likely contributory in the particular context of Polynesia. For example, with 5–10 percent of its total population being of Chinese origin, it is believed that the spirits of deceased people who died by hanging haunt the living, seeking revenge in Chinese culture. It is possible that similar cultural beliefs exist in Polynesian culture, but no data has been found on this specific topic.

Importantly, we found the vulnerability of SA to catastrophe like COVID-19 is not limited to the mentally ill. About half of our study subjects did not carry any psychiatric diagnosis (Table 3). In our study, the proportion of subjects with no psychiatric diagnoses was significantly higher (49.6%) in comparison to study of 2008–2010 (14.5%)^5^ (OR = 5.8 CI [4.3, 7.8], p < 0.001). This suggests a higher representation of SA linked to acute stressors or environmental difficulties of pandemic isolation, again, especially in younger groups during COVID era (63.9% of age less than 20 did not have a psychiatric diagnosis). This speaks for the utmost priority to prevent and mitigate the psychological sequela from catastrophe like pandemic, war, and climate change in general population, not just for patients with psychiatric disorder. The underlying complexity interaction between socioeconomical changes and disaster like pandemic underscores the need for appropriate and updated monitoring systems and research endeavor in real time. Our findings could help inform the development of prevention and intervention strategies for SA during the time of catastrophe. In the future, the strategy and policy of the crisis prevention and intervention should be applied to general population. The strategy of suicide prevention during disaster period like COVID-19 should be different from “regular” time that weighs more on mental illness-related SA.

Our study also found a slightly lower rate of recurrence (11.3% in 36 months) compared to other studies with a reported rate ranging from 12.4% to 16.0% in a 12 months interval.^19^^,^^20^ Nonetheless, a local study designed to explore the protective effects of traditional massage on suicide behaviors in Tahiti reported recurrence rate ranging from 3.3% to 12% at 12 months, which is more in line with our findings,^21^ and which may hint to a resilience of the population, the nature of SA during COVID-19 and the relative effectiveness of local support programs such as the Non-Governmental Organization SOS Suicide hotline. Strong firearms control is also an effective means to prevent SA in French Polynesia, with no SA recorded with this method. Restricting access to certain medication classes, such as painkillers, could be a possible strategy for addressing the high prevalence of auto-intoxication but would have inherent limitations in terms of overall prevention. As defined by Jean Benoist,^22^ the Polynesian archipelago exhibits strong characteristics of insularity, such as limited size and geographic isolation. These factors may play a dual role, resulting in intense interpersonal relationships, difficulties maintaining anonymity, and a need for increased organization into cultural or social subgroups. This can provide close interpersonal support and strong bonds between individuals when needed, but may also lower the crisis threshold in case of a dysfunctional relationship system whereas catastrophe like pandemic can add oil to the fire. Nevertheless, the relationship between sociostructural changes and SA facing catastrophe remains to be investigated. Also, future study shall consider emotional triggers or certain socio-demographic parameters such as education level, income and poverty, professional or marital status, which are known to be determinants of suicidal risk for SA.

In the present study, it is important to note that low rates of psychiatric comorbidities were found in the general cohort compared to what could be expected from other studies (Table 3). This apparently is due to the fact that nearly half of the cohort did not carry psychiatric diagnosis that “dilute” the rate of the psychiatric disorders. The low rate of depression (9.9%), for example, can also be explained by cultural and linguistic aspects. The overall prevalence of unipolar depression in French Polynesia has not been well assessed, except for the MHGP study,^2^ which recently reported rates ranging from 8.5% to 18.2% in the general population. The Non-Fatal Suicide Behaviors study conducted in 2016^5^ reported a rate of 45% for mood disorders, but it did not differentiate between subtypes, including bipolar disorder. However, based on clinical practice and in comparison to European countries, it is believed that the prevalence of unipolar depression is relatively low in French Polynesia. One possibility is that the equivalent of depression in the Polynesian culture may escape the classical diagnosis criteria and manifest through other dimensions, such as difficulties in maintaining impulse control. This could also explain the relative low proportion of diagnosed patients with depressive disorders in our sample, suggesting the presence of underdiagnosis. This topic has been explored in another publication by S. Amadeo regarding mental illness representations in French Polynesia.^23^

This study is subject to limitations as it only includes SA that received psychiatric evaluation. The study acknowledges that due to the significant geographic dispersion of the population and limited access to healthcare in general, a proportion of SA may not have been medically addressed and thus not included in the data collection. This can lead to an underestimation of SA rates, especially for demographic areas such as the archipelagos, and reduce the statistical power of our study for these subgroups. Amadeo et al.^4^ had identified this limitation and estimated the exclusion rate to be around 20%. However, the CHPF is the sole crisis referral center in French Polynesia with comprehensive outreach programs. The likelihood of “under-reporting” of SA is much lower than an urban setting with complex health care system in the rest of the world. Also, it is important to note that in our study, we did not have the exact pre-COVID incidence levels of SA for direct comparison, such as data from 2019. This makes it difficult to isolate the potential effects of the pandemic as distinct from the ongoing sociocultural impact preceding the pandemic. Furthermore, it is worth mentioning that our study did not include a proper follow-up period yet. This limitation can impact the interpretation of the repetition rate of SA and also limit our understanding of the long-term trajectory and outcomes.

Additionally, we did not consider individual parameters such as anthropologic characteristics, socio-economic status, marital status, religious practice or specific triggering events, which are well-known determinants of suicidal behavior and could have provided further and more qualitative insights into the results. Diagnosis evaluation was based on a predetermined semi-structured interview to reach diagnosis and characteristics of the suicide attempt, backed with medical records. While being a more time-efficient method compared to a structured diagnosis tool, it can expose in some extent to more confirmation bias during data collection.

In conclusion, our study has the advantage of applying a similar methodology in the same setting a decade apart and also allowing for a prospective analysis during a critical period of catastrophe. Our study has revealed several critical findings that can enrich our understanding of the suicidal behavior during catastrophe. First, there has been a notable 54.9% increase in the incidence of SA during the third year of COVID-19 compared to the levels observed in 2010, presumably due to the detrimental stress COVID-19 has brought. Second, this increase has exhibited a consistent and significant upward trend over the course of the 36-month period during COVID-19 pandemic. Additionally, we have observed that a significant proportion of SA cases are not associated with mental illness, highlighting the complexity of biopsychosocial factors contributing to the SA during catastrophe. Moreover, our findings have identified a higher risk of SA in the urban region of French Polynesia, Tahiti, as well as among young adults and in particular teenage women.

In light of the global uncertainty caused by the COVID-19 pandemic and its disruptive effects on social structures, it is important to continue monitoring suicide behaviors and mortality rates in the coming years, as the psychological consequences of the pandemic may manifest over the long term whereas central nervous system involvement of long COVID is also evident. Without a doubt, factors such as limited medical resources, socioeconomic disparities, and social isolation could contribute to increased mental health risks and SA behaviors.^24^ In the near future, we aim to enrich our findings by perpetuating local monitoring systems on the epidemiology of SA in French Polynesia, as well as exploring the determining factors of suicide through complementary studies, such as the AUTOPSOM program.^25^ While our study primarily focuses on the quantitative aspects of suicide attempts, the current conjunction with the COVID-19 pandemic and the intriguing results we obtained since the last study also highlight the need for a more qualitative and individual-based approach. Factors such as political perception and change, growing economic disparities within the population, social isolation, impact on psychotraumatic history and vulnerability, grief, and direct exposure to COVID-19 (strains) are all specific factors we would like to investigate in future study. To prevent SA, promoting mental health, launching prevention campaigns, establishing a local observatory, providing a suicide prevention and intervention program, and involving healthcare professionals, policymakers and social organizations are recommended. Additionally, ongoing anthropocultural research on the determinants of the outcome from catastrophe and access to care mitigation should be pursued. We believe the findings of this study have important implications for crisis prevention and intervention strategies and policies in French Polynesia and other vulnerable societies facing catastrophes like pandemics, war, and climate change.

## Contributors

JS: study design, investigation, statistical analysis, writing of the manuscript.

MS: data curation and collection, review & editing.

VC: data curation and collection, review.

GET: formal analysis and interpretation, review and writing of the manuscript.

## Data sharing statement

Once published, primary data and analysis outcomes will be stored in a securely active database within the CHPF for up to two years after publication. Subsequently, they will be transferred to an intermediary database for a maximum period of twenty years. The determination of this retention timeframe follows the guidelines of the CNIL (Commission Nationale de l’Informatique et des Libertés) and is subject to assessment by the Data Protection Officer (DPO).

Access to the data and analytical files utilized in our study is strictly prohibited for any individuals or authorities without explicit permission from both the principal author and the authorized representative of the French Polynesia Hospital Centre.

## Declaration of interests

G.E. TSAI holds equity and is CEO and Chair of Board of SyneuRx, developing novel antidepressants.

## Appendix A Supplementary data

Translated Abstract with Disclaimer
