# Supplementary material for: Suicide attempts in French Polynesia during the era of COVID-19: a prospective analysis over three years
Source: Lancet Reg Health West Pac. 2023 Sep 4;42:100899. doi: 10.1016/j.lanwpc.2023.100899 (PMC10865017; doi:10.1016/j.lanwpc.2023.100899)
Supplement: Translated Abstract with Disclaimer [file mmc1.docx]

**RESUME**

**Contexte :** Des études antérieures en Polynésie française ont identifié les tentatives de suicide comme un problème préoccupant, avec un taux d'incidence annuel mesuré de 79,4 tentatives pour 100 000 habitants entre 2008 et 2010. En réponse à la pandémie de COVID-19, un système de suivi et d'enquête sur les tentatives de suicide (TS) a été mis en place.

**Méthodes :** Une étude prospective a été menée entre avril 2020 et mars 2023, incluant tous les patients adressés au Centre Hospitalier de Polynésie française pour TS. Les facteurs démographiques ainsi que les paramètres cliniques ont été analysés.

**Résultats :** Au cours de la période d'étude, 895 TS ont été enregistrées et confirmées, avec un taux brut annuel de 106,7 événements et un taux ajusté de 113,2 pour 100 000 habitants. La grande majorité des TS se sont produites sur l'île de Tahiti. La moitié des sujets n'avaient pas de diagnostic psychiatrique. Une augmentation significative des TS a été observée entre la première et la troisième année, avec une vulnérabilité particulière des jeunes (surtout les femmes) à Tahiti. L'incidence normalisée chez les femmes de moins de 20 ans a atteint 310,4 pour 100 000 habitants.

**Interprétation :** Nos données ont révélé une augmentation globale de 34,4 % des TS en Polynésie française, avec une hausse marquée de 54,9 % au cours de la troisième année de la pandémie. Le taux d'incidence record de la dernière année est confirmé par une augmentation du recours aux lignes d'assistance téléphonique pour le suicide, notamment à Tahiti. Une corrélation entre l'exposition à la COVID-19 et les comportements suicidaires, tant au niveau individuel que social, est suspectée, en particulier chez les jeunes femmes à Tahiti. Ces résultats soulignent la nécessité de renforcer la prévention et de maintenir un système de suivi efficace des tentatives de suicides même après la fin de l'état d'urgence sanitaire.

***This translation in French was submitted by the authors and we reproduce it as supplied. It has not been peer reviewed. Our editorial processes have only been applied to the original abstract in English, which should serve as reference for this manuscript.***
